# Post-Operative Thoracic Drainage

**Published:** 1919-01

**Authors:** 


***Post-Operative Thoracic Drainage.*** By WILLIE MEYER, M. D. *Annals of Surgery*, August, 1918.

The author concludes in the following way a long article dealing with thoracic drainage:

1) Thoracic operations, like operations in other parts of the body, often demand drainage.

2) With no adhesions present between the two pleural leaves, an acute pneumothorax is the inevitable consequence if an ordinary drain is introduced. The occurrence of a complete pneumothorax after operation greatly increases the dangers confronting the patient during the after-treatment.

It is, therefore, necessary to avoid this complication. This could hitherto be accomplished either by leaving the patient under the influence of differential air-pressure for a greater part of the first 24 hours following the operation, having closed the thoracic wound, and then closed the drain ends outside with a large piece of rubber dam (Willie Meyer), or by making use of Tiegel's metallic drain. Both methods have been tried and found satisfactory, though both have certain drawbacks.

3) Kenyon's method of draining empyema in children has been found to be useful also for post-operative drainage in general. It is simple, safe, and easy of execution. It permits of satisfactory examination of the quality and quantity of the effused fluid.

Kenyon's method of post-operative drainage fulfils Sauerbruch's demand that the thorax be closed air- and water-tight after intrathoracic operations. Yet it permits of draining off, in an efficient manner, the secretions of the pleura, which follow the majority of intrathoracic operations, and usually are not sterile.

5) The introduction of Kenyon's method of drainage, therefore, bids fair to be a long step forward in the evolution of thoracic surgery. It greatly adds to the safety of intrathoracic surgical work, and should, for the present at least, be employed *after every operation* upon the thorax in which the free pleural cavity has to be traversed.

Summarizing, it may be said that, at the present moment, the successful issue of surgical work within the thorax seems best assured by combining immediate complete closure of the incision with an efficient method of simple and safe drainage of fluid and air, without allowing the latter to regurgitate into the chest.

